# Predictive Modeling of Coral Disease Distribution within a Reef System

**DOI:** 10.1371/journal.pone.0009264

**Published:** 2010-02-17

**Authors:** Gareth J. Williams, Greta S. Aeby, Rebecca O. M. Cowie, Simon K. Davy

**Affiliations:** 1 School of Biological Sciences, Victoria University of Wellington, Wellington, New Zealand; 2 Hawaii Institute of Marine Biology, Kaneohe, Hawaii, United States of America; University of North Carolina at Chapel Hill, United States of America

## Abstract

Diseases often display complex and distinct associations with their environment due to differences in etiology, modes of transmission between hosts, and the shifting balance between pathogen virulence and host resistance. Statistical modeling has been underutilized in coral disease research to explore the spatial patterns that result from this triad of interactions. We tested the hypotheses that: 1) coral diseases show distinct associations with multiple environmental factors, 2) incorporating interactions (synergistic collinearities) among environmental variables is important when predicting coral disease spatial patterns, and 3) modeling overall coral disease prevalence (the prevalence of multiple diseases as a single proportion value) will increase predictive error relative to modeling the same diseases independently. Four coral diseases: *Porites* growth anomalies (PorGA), *Porites* tissue loss (PorTL), *Porites* trematodiasis (PorTrem), and *Montipora* white syndrome (MWS), and their interactions with 17 predictor variables were modeled using boosted regression trees (BRT) within a reef system in Hawaii. Each disease showed distinct associations with the predictors. Environmental predictors showing the strongest overall associations with the coral diseases were both biotic and abiotic. PorGA was optimally predicted by a negative association with turbidity, PorTL and MWS by declines in butterflyfish and juvenile parrotfish abundance respectively, and PorTrem by a modal relationship with *Porites* host cover. Incorporating interactions among predictor variables contributed to the predictive power of our models, particularly for PorTrem. Combining diseases (using overall disease prevalence as the model response), led to an average six-fold increase in cross-validation predictive deviance over modeling the diseases individually. We therefore recommend coral diseases to be modeled separately, unless known to have etiologies that respond in a similar manner to particular environmental conditions. Predictive statistical modeling can help to increase our understanding of coral disease ecology worldwide.

## Introduction

The notion of a complex web of interactions between a disease and its environment has been postulated for centuries [1] and stems from the fact that diseases often have intricate etiologies [2] and different modes of pathogen transmission between hosts [3]. Furthermore, pathogen virulence can respond positively or negatively to a range of variables, such as temperature, nutrient availability, or habitat quality [4]–[6]; changes in environmental conditions can promote physiological stress that impairs host immunity [7]–[9], and there may be differences in disease susceptibility between host genotypes [10], [11]. With this in mind, it is easy to envisage how complex associations between a disease, the host, and the environment can become established. For example, cholera in humans is caused by *Vibrio cholerae*, a bacterium that attaches to zooplankton[12]. Outbreaks of cholera are not the result of changes in a single environmental factor, but instead involve multiple interactions between human host densities, *V*. *cholerae*, water temperature, salinity, and copepod abundance, and are generally a result of zooplankton blooms following heavy rainfall [13].

Marine organisms are also subject to the influence of disease. Coral reefs worldwide are in decline [14]–[16] and the role of marine diseases, in particular coral disease, to this decline is receiving increasing attention [5], [17]–[20]. Coral disease outbreaks can lead to an overall reduction in live coral cover [21] and reduced colony density [22], and in extreme cases initiate community phase-shifts from coral- to algal-dominated communities [23]. Coral diseases can also result in a restructuring of coral populations [24], for example a shift from long-lived slow growing massive reef builders to communities dominated by smaller, shorter-lived corals [25]. As corals act as facilitators for other reef invertebrates [26] and fish [27] their loss threatens coral reef biodiversity and function. Spatial patterns of coral disease are linked to environmental conditions [28]. Significant relationships exist between coral disease prevalence and elevated water temperatures [29]–[32], a decline in water quality [33]–[37], vector and host densities [31], [38], and intensity of coral bleaching [39], [40]. The effects of environmental factors on coral disease prevalence and modes of transmission are likely to be intricate and synergistic [41]. Recently, efforts have shifted towards this multi-factorial concept. For example, scleractinian coral white syndrome outbreaks along the Great Barrier Reef (GBR) require a threshold coral cover of greater than 50% in conjunction with thermal stress events, and the interaction between the two predictors explains a significant amount of the increase in the frequency of outbreaks [31]. In Kenya, the relationship between massive *Porites* growth anomaly prevalence and 16 environmental parameters including water quality, temperature, intensity of bleaching, and benthic composition were modeled to reveal bleaching intensity as the most important factor in explaining spatial distribution of the disease [40]. However, researchers and monitoring programs are still, on occasion, attempting to understand spatial patterns of overall coral disease prevalence (combining the prevalence of multiple diseases into a single proportion value as the response variable) with the environment. This approach ignores the common-sense notion that diseases with different pathogens and hosts are unlikely to have common spatial/temporal patterns or environmental associations, and therefore should be monitored and analyzed individually unless known to have a similar cause.

Exploring coral disease spatial patterns requires a statistical technique that effectively addresses the complexity of disease ecology, in particular the potential for non-linear relationships between the disease, host and environment [42]. One approach is classification and regression tree (CART) modeling [43]. Regression trees have several advantages as a modeling technique, including that various types of predictor and response variables can be analyzed simultaneously rather than in an iterative manner, missing values in data sets can be incorporated and therefore information loss minimized, and complex interactions between predictors can be quantified and modeled in a simple manner [44]. Despite these advantages regression trees are often poor predictors and large trees can be difficult to interpret [44]. Recently these weaknesses have been overcome with the use of boosted regression trees (BRT) [44]–[49], which incorporate machine learning decision tree methods [50] and boosting, a method for improving model accuracy (reducing predictive error) [46]. BRT differs fundamentally from conventional techniques that aim to fit a single parsimonious model. Instead, the final BRT model is an additive regression model in which individual terms are simple trees, fitted in a forward stage-wise manner [46]. In summary, BRT gives two crucial pieces of information, namely the underlying relationship between the response and each predictor, and the strongest statistical predictor (among the simultaneously tested predictors) of the response in question. These are clearly two different things, and as BRT focuses on building predictive models for theory development, the environmental associations that result can be direct or indirect. Disease-environment relationships revealed by this type of modeling can be used to predict spatial patterns in novel systems and facilitate hypothesis-driven experimental studies. Exploratory and predictive modeling provides an initial step towards understanding spatial patterns and processes and has been underutilized in coral disease research.

In the present study, we used a BRT technique and a reef system with contrasting environmental conditions and a range in coral disease states and prevalence to address the following hypotheses: 1) coral diseases show distinct associations with multiple environmental factors, 2) incorporating interactions (synergistic collinearities) among environmental variables is important when predicting coral disease spatial patterns, and 3) modeling overall coral disease prevalence (the prevalence of multiple diseases as a single proportion value) will increase predictive error relative to modeling the same diseases independently. In addition, to develop the use of BRT modeling in coral disease research we outline the analytical methods of a technique and its novel application.

## Materials and Methods

### Model System, Host Sensities and Disease Prevalence

**Figure 1:**
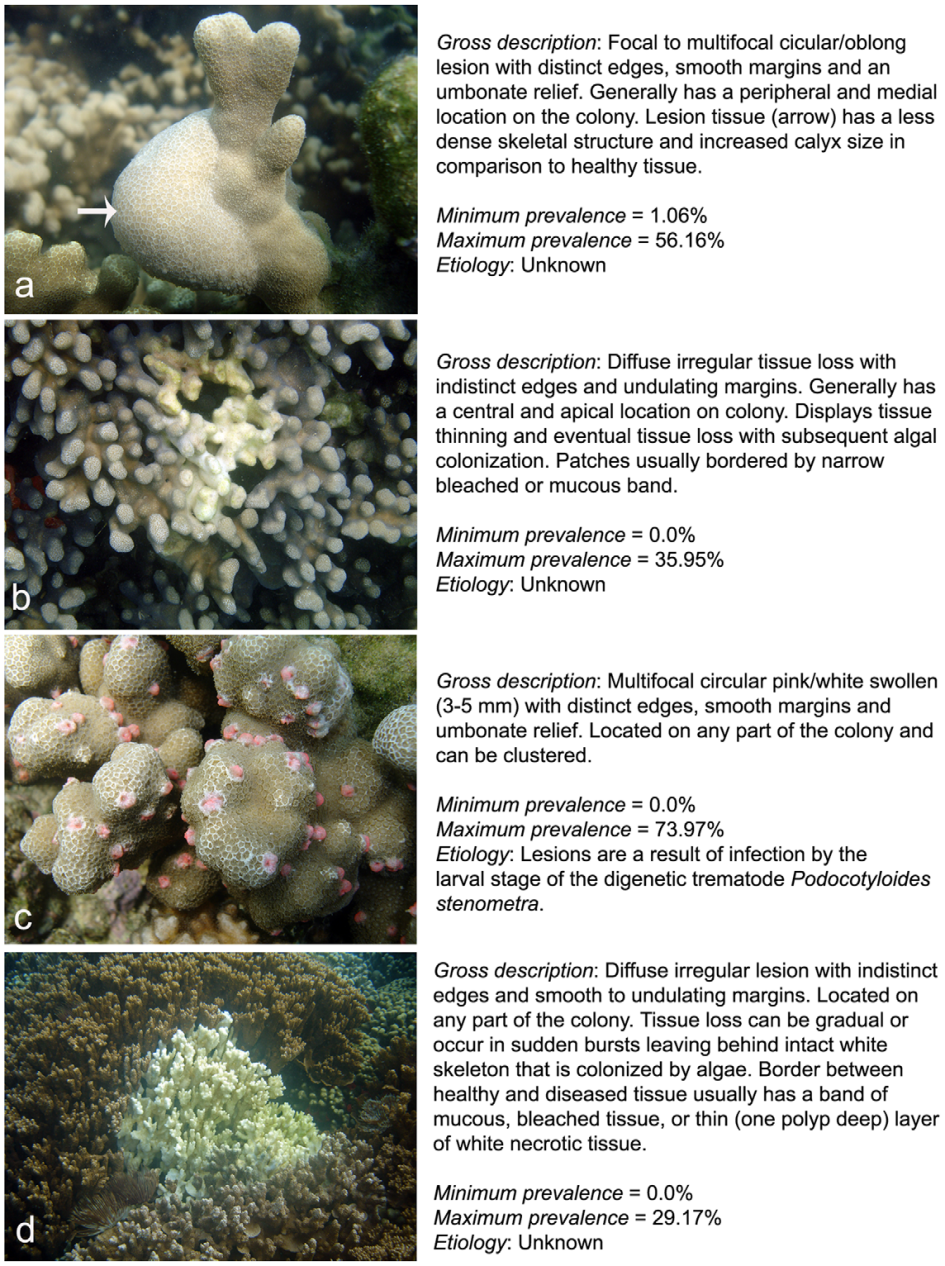
Gross descriptions of the four coral diseases present at Coconut Island Marine Reserve, Oahu, Hawaii. a) *Porites* growth anomaly, b) *Porites* tissue loss, c) *Porites* trematodiasis, and d) *Montipora* white syndrome. Minimum and maximum prevalence values between transects are shown.

In August 2007, pilot surveys were conducted within Coconut Island Marine Reserve (CIMR) (21°26′N, 157°47′W), Kaneohe Bay, Oahu, Hawaii. The two competitively dominant space holders in the system were *Porites compressa* and *Montipora capitata*. *Pocillopora damicornis*, *P. meandrina* and *Fungia scutaria* were also observed but at low densities. Four disease states affecting *Porites* and *Montipora* spp. were documented and CIMR was found to represent an ideal system for modeling coral disease-environment associations due to large variations in host abundance, disease prevalence, and environmental conditions over spatial scales of 100s m (Fig. 1, Table 1).

**Table 1:** Predictor variables used in the analyses with their codes and units.

Variable	Type	Code	Description and units	Min	Max	Range
temperature	environmental	Temp	°C	23.0	27.3	4.3
salinity	environmental	Sal	ppt	31.30	35.3	4.0
turbidity	environmental	Turb	standard turbidity units (STU)	2.15	9.69	7.5
chlorophyll-*a*	environmental	Chl-*a*	µg/l	0.25	1.04	0.8
depth	environmental	Depth	m	0.74	3.06	2.3
sedimentation	environmental	Sed	g/m^2^/day	27.7	89.8	62.1
organics	environmental	Org	% of sediment	3.7	12	8.3
*Porites* cover	biological	*Porites*	%	9	68	59
*Porites* density	biological	PorDen	number of colonies/m^2^	1.5	15	13.5
*Montipora* cover	biological	*Montipora*	%	2	42	40
*Montipora* density	biological	MonDen	number of colonies/m^2^	1.1	33.4	32.3
total coral cover	biological	Cover	%	28	87	59
total coral density	biological	Den	number of colonies/m^2^	5	12	7
juvenile parrot fish	biological	JuvPF	number per 300 m^2^	4	489	485
butterflyfish density	biological	BF	number per 300 m^2^	0	13	13
reef type	categorical	Reef	upper slope *versus* reef flat	−	−	−
season	categorical	Season	first *versus* second season	−	−	−

Min/Max, minimum and maximum predictor values between transects.

We conducted surveys over two five-week periods: October – November 2007 (winter), and May – July 2008 (summer). The sampling design was not hierarchical, but instead was designed to maximize variability between observations in both disease prevalence and the environmental predictors. Observations were randomized within 11 specific regions of CIMR known, from preliminary surveys, to display contrasting disease prevalence and environmental conditions. To quantify disease prevalence, 55 belt transects (10×2 m) were surveyed within a depth range of 0.7–3.1 m in each season (giving 110 observations overall). Lesions on colonies were classified according to gross morphology (growth anomalies, tissue loss, discoloration, trematodiasis) and assigned the host genus and descriptive name [51] (Fig. 1). *Porites* trematodiasis (PorTrem) was recorded even if a single lesion was found on a colony. The proportion of diseased colonies was calculated for each disease and the overall (total) disease prevalence. To quantify host abundance, every coral colony whose centre fell within the belt transect area was counted and inspected for signs of disease. Percentage cover of live coral was estimated using a point-intercept method at 50-cm increments along the transect line.

### Environmental and Biological Predictors

Salinity, turbidity and chlorophyll-*a* were measured using two RBR® XR-420 data loggers (www.rbr-global.com) recording every minute over 24 hr periods at the depth of the coral. The chlorophyll-*a* value is a measure of how much of the suspended material present (turbidity) contains chlorophyll-*a*. The placement of the loggers was randomized among the 11 CIMR regions throughout each 5-week period. HOBO® Pro temperature data loggers (www.onsetcomp.com) were attached to the reef within each of the 11 regions; these recorded every 10 min from the start of October 2007 to the end of July 2008.

Sedimentation levels were measured as a potential source of stress to the corals which in turn may influence their susceptibility to disease. Within each of the 11 regions, sedimentation was quantified using PVC sediment traps [52]. These were attached to stainless steel poles and placed into, and approximately 30 cm above, the substrate among coral colonies. Sedimentation was measured over 7-day periods, with measurements repeated 5 times per season. To determine the organic carbon fraction of the sediment (a proxy for the level of organics, but not dissolved organics, entering the system), sediment was finely ground, oven dried at 100°C for 10 h and weighed. Samples were then placed in a muffle furnace at 500°C for 12 h to burn off the organic fraction and the remaining inorganic fraction reweighed [53].

Physical injury to the host coral can promote the spread of some coral diseases [54]. Reef fish, such as butterflyfishes, parrotfishes and damselfish, offer a potential source of injury to corals [55]–[57] and fish are known to be involved in disease transmission [58] and/or promoting the rate of disease spread [59]. Within CIMR, pilot surveys showed butterflyfish to be the dominant coral-feeding fish. Damselfish and adult parrotfish were seldom seen but juvenile parrotfish were abundant and parrotfish feeding scars were seen around CIMR, particularly on *P. compressa*. Hence, only coral-feeding butterflyfish (facultative and obligate) and juvenile parrotfish were quantified over a 50×6 m area within the vicinity of each disease transect. The observer swam at a speed of approximately 10 m min^−1^ to account for the active nature of these reef fish and 1 m out from the reef-flat edge to detect fish both on the reef flat and slope. Horizontal visibility limited the width of the transect, with 3 m being the limit at which fish could confidently be identified to species. Butterflyfish species observed were *Chaetodon auriga*, *C. ephippium*, *C. lineolatus*, *C. lunula*, *C. lunulatus* (formally *C. trifasciatus*), *C. multicinctus*, *C. ornatissimus*, and *C. unimaculatus*. Each count was conducted between the daylight hours of 10:00 and 15:00 and replicated a minimum of five times, with each count taking place on a different day.

### Statistical Analysis

The 110 belt transects (55 from each season) were modeled simultaneously against 17 predictor variables, which included continuous environmental data, count data, and categorical terms (Table 1). Transects were considered independent observations in the models, and not pseudoreplicates, as they were separate from each other in both space and time. We used Boosted Regression Trees (BRT) [46] as the modeling technique. The technique is sometimes referred to as stochastic gradient boosting, as BRT includes an element of stochasticity in order to improve accuracy and reduce overfitting (when a statistical model describes random error or noise instead of the underlying relationship) [60]. BRTs were constructed using the routines *gbm* version 1.5–7 [61] and *gbm.step* [46] in the R statistical program version 2.6.2 (R Development Core Team, http://www.r-project.org). Prevalence data was log transformed and the few zero disease prevalence counts that did occur removed in order to achieve a normal/pseudo-normal distribution. The numbers of independent observations were then as follows: *Montipora* white syndrome (n  =  101), *Porites* trematodiasis (n  =  86), *Porites* tissue loss (n  =  101), *Porites* growth anomalies (n  =  110), and overall disease (n  =  110). Analyses were based on a Gaussian distribution. Due to problems with assigning real probabilities in BRTs (there are no *p*-values) a key approach is to use validation processes that require a proportion of the data set to be held back. Due to our relatively small data set, we used 10-fold cross-validation (cv) for model development and validation, with the benefit of still using the full data set to fit the final model. The measure of model performance was cv deviance and standard error (se) throughout our study (the lower the value the better the model performance). Within the BRT model, three terms are used to optimize predictive performance: bag-fraction, learning rate, and tree complexity. The bag-fraction determines the proportion of data to be selected at each step and therefore the model stochasticity; for example a bag fraction of 0.5 means that 50% of the data are drawn at random without replacement. The learning rate (lr) is used to shrink the contribution of each tree as it is added to the model, and tree complexity (tc) determines the number of nodes in a tree and should reflect the true interaction order on the response being modeled [62]. We determined optimal settings for these parameters by examining the cv deviance over tc values 1–5, lr values of 0.05, 0.01 and 0.001, and bag fractions of 0.5 and 0.75. All possible combinations were run, with the optimal number of trees in each case being determined by *gbm.step*. The combination of the three parameter settings with the lowest cv deviance was then selected to produce the final BRT. Finally, redundant predictor variables may degrade model accuracy by increasing variance, particularly in small data sets. We therefore achieved optimization to create a balance between statistical performance, parsimony, and usefulness of the model using the routine *gbm.simplify*, a method analogous to backwards selection in regression [46]. Both season and reef type (categorical predictors) were found to exert no influence upon predicting the prevalence of any disease and were removed during optimization before the creation of the final BRTs.

As part of the final model, BRT assesses the relative importance (or contribution) of each predictor. This measure is based on the number of times a variable is selected for splitting, weighted by the squared improvement to the model as a result of each split, and averaged over all trees [45], [46]. A higher relative importance of a predictor indicates a stronger influence on the response (disease) in question. Partial dependency plots were used for interpretation and to quantify the relationship between each predictor variable and the disease, after accounting for the average effect of all other predictor variables in the model. To quantify interaction effects between predictors (the collinearity and synergistic effect upon predicting the response in question) we used the routine *gbm.interactions* [46]. The relative strength of interaction fitted by BRT is quantified by the residual variance from a linear model, and the value indicates the relative degree of departure from a purely additive effect, with zero indicating no interaction effects fitted. The interaction value can also be thought of as the relative contribution of the interaction between the two predictors towards the overall predictive performance of the individual model (the cv deviance value). We defined a threshold interaction value and reported the interactions with values ≥ 0.1. We performed the above analyses for individual diseases and for the calculation of overall disease prevalence.

## Results

### Environmental Associations and Strongest Predictors

#### Porites growth anomalies (PorGA)

**Figure 2:**
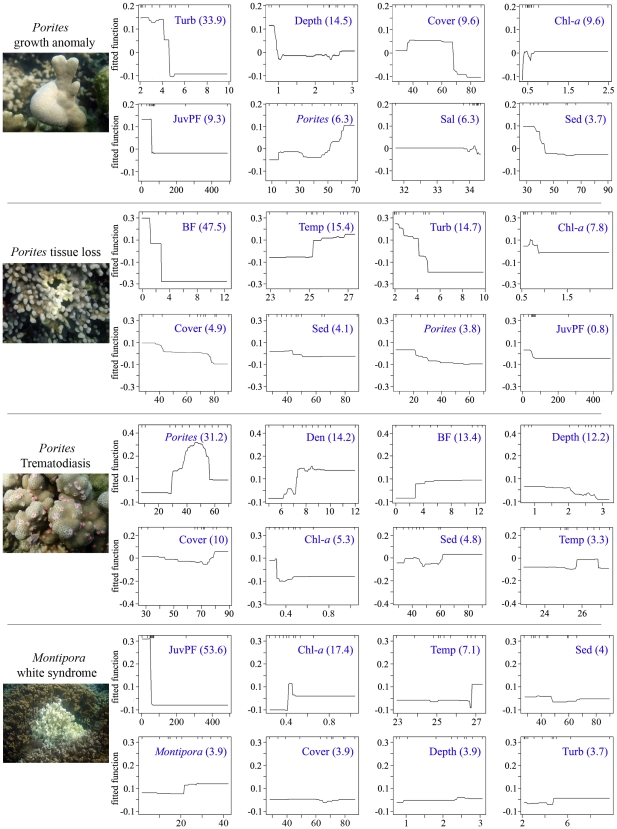
Boosted regression tree (BRT) analyses relating prevalence of four coral diseases to environment. Models are developed and validated using 10-fold cross-validation on 86–110 independent observations for each disease and 17 predictor variables. The 8 most influential predictors to the model are shown. Their relative importance is shown as a % in parentheses. The deciles of the distribution of the predictors are indicated by tick marks along the top of each plot. Predictor variable codes and units are as per Table 1.

Two relationships contributed most strongly to predicting PorGA prevalence (Fig.2), namely negative relationships with both turbidity and depth. PorGA prevalence was highest in clearer waters within 1 m of the surface. In addition, prevalence peaked when there was an overall coral cover of 40–70%, increased chlorophyll-*a* concentration within any suspended material, lower juvenile parrotfish abundance, and an increase in *Porites* cover. Turbidity offered the largest contribution (i.e. it was the strongest predictor) with a relative importance of 33.9% (Fig.2). Model cv deviance equalled 0.391, with second order interactions present between predictors (Table 2). The largest interaction (collinearity and synergistic effect) involved *Porites* cover and total coral cover (Table 3).

**Table 2:** Optimal settings and predictive performance of boosted regression tree (BRT) analyses relating prevalence of four coral diseases to environment.

Disease	number of trees	lr	tc	bag fraction	cv deviance	se
*Porites* growth anomaly	3150	0.01	3	0.75	0.391	0.02
*Porites* tissue loss	1950	0.01	3	0.75	0.350	0.01
*Porites* trematodiasis	4400	0.01	4	0.75	1.182	0.14
*Montipora* white syndrome	1700	0.01	3	0.75	0.213	0.04
Overall disease prevalence	2550	0.01	3	0.5	3.215	1.26

lr, learning rate; tc, tree complexity. Cross-validation (cv) deviance and standard error (se) is shown as the measure of model performance (the lower the value the better the model performance).

**Table 3:** Pairwise interactions between predictor variables used to relate prevalence of four coral diseases to environment.

Disease	Predictor	Predictor	Interaction Value	Pairwise interaction summary
*Porites* growth anomaly	*Porites* cover	Total coral cover	0.86	Higher *Porites* cover (>60%) and high total coral cover (40–70%).
	Chlorophyll-*a*	Turbidity	0.32	Higher chlorophyll-*a* and lower turbidity.
	Juvenile parrotfish	Sedimentation	0.30	Lower juvenile parrotfish abundance and lower sedimentation.
*Porites* tissue loss	Butterflyfish	Turbidity	0.21	Lower butterflyfish abundance and lower turbidity.
	*Porites* cover	Turbidity	0.14	Lower *Porites* cover (<20%) and lower turbidity.
	*Porites* cover	Temperature	0.10	Lower *Porites* cover (<20%) and higher temperatures (>25°C).
*Porites* trematodiasis	*Porites* cover	Total colony density	2.02	Mid *Porites* cover (50%) and higher total colony density (>7/m^2^).
	Total colony density	Chlorophyll-*a*	0.95	Higher total colony density (>7/m^2^) and lower chlorophyll-*a*.
	*Porites* cover	Chlorophyll-*a*	0.74	Mid *Porites* cover (50%) and lower chlorophyll-*a*.
	*Porites* cover	Temperature	0.39	No clear association with temperature.
	Temperature	Depth	0.20	No clear association with depth.
	Total colony density	Temperature	0.11	No clear association with temperature.
*Montipora* white syndrome	Chlorophyll-*a*	Temperature	0.15	Higher chlorophyll-*a* and higher temperatures (>27°C).

Interactions displayed are those that exceeded an interaction value of ≥0.1 and involved the 8 predictors offering the highest contribution to the model displayed in Figure 2. Interaction value indicates the relative degree of departure from a purely additive effect, with a value of zero indicating that no interaction is present. A summary description is given for the association of the peak in disease prevalence and the pairwise interactions for those predictor variables showing a clear relationship (for example positive, negative, or modal) with the disease in Figure 2.

#### Porites tissue loss (PorTL)

Three relationships contributed most strongly to predicting PorTL prevalence (Fig.2): a negative correlation with butterflyfish abundance, a positive correlation with temperature, and a negative correlation with turbidity. Prevalence peaked in areas with few butterflyfish, where temperatures reached above 27°C, and low turbidity environments. Butterflyfish abundance was the strongest predictor with a relative importance of 47.5% (Fig.2). Model cv deviance equalled 0.350, with second order interactions present between predictors (Table 2). The largest interaction involved butterflyfish (the strongest predictor) and turbidity (Table 3).

#### Porites trematodiasis (PorTrem)

Four relationships contributed most strongly to predicting PorTrem prevalence (Fig.2). A modal relationship occurred with *Porites* cover, with a peak in prevalence at approximately 50% cover, and a positive correlation existed with overall colony density, reaching an asymptote at approximately 9 colonies m^2−1^. There was a positive correlation with butterflyfish abundance (peaking above 4 fish 300 m^2−1^), and a weak negative correlation with depth (Fig.2). *Porites* cover was the strongest predictor with a relative importance of 31.2% (Fig.2). Model cv deviance equalled 1.182, the highest deviance for any of the individual models, with third order interactions present between predictors (Table 2). The largest interaction involved *Porites* cover and overall colony density (the two strongest predictors). This was the largest interaction value (2.02) seen within any of the models (Table 3).

#### Montipora white syndrome (MWS)

Two relationships contributed most strongly to predicting MWS prevalence, namely a negative correlation with juvenile parrotfish abundance and a positive correlation with chlorophyll-*a* concentration (Fig.2). In addition, a positive correlation existed with temperature, with peak prevalence occurring above 27°C. Juvenile parrotfish abundance was the strongest predictor with a relative importance of 53.6% (Fig.2). Model cv deviance equalled 0.213, the lowest deviance (best fit) for any of the models, with second order interactions present between predictors (Table 2). The single interaction involved chlorophyll-*a* with temperature (Table 3). This was the only model where the strongest predictor (juvenile parrotfish abundance) did not interact with another predictor variable above the defined interaction threshold.

#### Combining disease states (overall prevalence)

Combined modeling of the diseases led to a loss in predictive performance (increased predictive error) of the model, with an approximate six-fold increase in cv deviance above the average cv deviance for all four diseases analysed individually (Table 2).

## Discussion

Coral diseases, like most diseases, can logically be expected to display complex associations with their environment due to the intricate nature of the host -environment-pathogen triad of disease causation [2], and the inherent multi-collinearity present between biotic and abiotic variables in any ecological system. With the use of boosted regression tree (BRT) modeling, this study has shown that different coral diseases do indeed show complex associations with a range of environmental variables and that these associations are distinct between diseases. We determined the environmental associations, and of these, the strongest statistical predictors of four individual coral diseases within a reef system in Hawaii from a suite of 17 predictor variables. The environmental conditions showing the strongest overall associations (direct or indirect) with coral disease prevalence in our model system were fish abundance, host availability, temperature, water quality (turbidity and chlorophyll-*a* concentration), and depth.

### Biotic, Abiotic and Physical Associations with Disease

Within our study the relative importance of disease associations (direct or indirect) with biotic, abiotic and physical parameters differed across coral disease states. *Porites* growth anomalies (PorGA) were optimally predicted by turbidity (abiotic), *Porites* tissue loss (PorTL) and *Montipora* white syndrome (MWS) by a decline in reef fish abundances (biotic), whilst spatial patterns of *Porites* trematodiasis (PorTrem) were optimally predicted by *Porites* host cover (biotic). The ecological mechanisms behind these disease-environment patterns are likely to be complex. Reef fish could be involved in regulating the disease dynamics of PorTL and MWS directly by offering a mechanism for diseased tissue removal via predation that could lead to individual host recovery [63]. Conversely, the association could equally be indirect and overall conditions which have negative effects on butterflyfish and juvenile parrotfish abundance may favor PorTL and MWS prevalence. In the Philippines, negative relationships between coral disease prevalence and fish taxonomic diversity exist inside and outside of marine protected areas [64], and in Palau increased prevalence of skeletal eroding band disease is associated with a reduction in the richness of a fish species targeted by fishers [65]. Further research is needed to tease apart the direct and indirect associations between coral disease prevalence and reef fish.

In addition to reef fish, we found strong links between the spatial patterns of PorTrem and a further biotic predictor, namely host abundance. The relationship between disease prevalence and host abundance is central to the theory of disease ecology [3]. As transmission is a key process in host-pathogen interactions, increased host density can increase horizontal transmission of a disease [66]. Hence, to a degree, host availability can determine how many infected individuals are observed in a defined area [67], regulated by both density dependent [68]–[70] and frequency dependent processes [71]–[73]. Scleractinian coral white syndrome outbreaks along the Great Barrier Reef require, in part, an overall coral cover in excess of 50% [31]. Positive correlations between coral cover and prevalence of black band, yellow band and white band disease were reported at reefs in Dubai [74], and positive relationships between overall disease and *Porites* cover in the Philippines [41]. PorTrem is caused by a digenetic trematode that relies on trophic transmission for completion of its multi-host (fish, mollusc, coral) life cycle [75]. Infected coral polyps are fed upon by coral-feeding fish, such as butterflyfish, which then become infected with the adult worm. Transmission of PorTrem across the reef occurs when the parasite's eggs are shed with the fish host feces. It therefore follows that as *Porites* cover and colony densities increase the chance of infected feces landing on the *Porites* host also increases, hence the positive relationship. The reason for the drop at higher levels of *Porites* cover is unclear and has been found across the entire Kaneohe Bay area [38]. Additionally, PorTrem is unable to establish without the full compliment of intermediate hosts and therefore the positive relationship with butterflyfish abundance is not surprising. Increased butterflyfish abundance leads to more infected polyps being fed upon and in turn more infected feces dropping over the reef.

Disease spatial patterns are often predicted by abiotic as well as biotic parameters. Among our four coral diseases we found PorGA and PorTL were both associated with reduced water turbidity, PorTL was positively associated with temperature, and MWS was positively associated with water chlorophyll-*a* concentration. For PorGA, water turbidity and depth (the sole physical parameter) were superior to any of the biotic parameters in predicting the prevalence of the disease, with prevalence peaking in shallow, less turbid waters. Little is known about PorGA ecology but it has been speculated that growth anomaly formation in corals could be linked to increased sensitivity to ultraviolet radiation (UVR) [76], [77]. Improved water clarity and shallow depths (with subsequent low light attenuation) could lead to increased levels of UVR reaching the coral surfaces [78]. In addition, shallower depths are often associated with higher variations in environmental stressors such as temperature and salinity and these fluctuations may be affecting PorGA prevalence.

A positive association between disease prevalence and temperature, as seen with PorTL and to a lesser degree with MWS in our study, is common in disease ecology. Increased temperature, like any environmental stressor, can shift the balance in favour of either the host or pathogen [10]. Compromized hosts may be more susceptible to ubiquitous pathogens and/or the stressor may increase pathogen virulence [5], [7], [79]. For example, malaria prevalence is often associated with temperature. At higher temperatures the parasite development time inside the mosquito vector shortens and so mosquitoes become infectious sooner and transmission rates increase [80]. For corals, increased temperatures can lead to loss of the symbiotic algae (bleaching) and possible mortality [81]. Higher water temperatures can also promote bacterial growth [5]. For bacterial diseases, the combined effect of temperature stress on the coral host and enhanced bacterial growth may ultimately result in disease occurrence. This was recently found in the Virgin Islands where coral bleaching led to a lethal white plague disease outbreak [82]. Many coral diseases show positive associations with temperature, such as black band disease in the Caribbean [83], [84], the Florida Keys [85], the GBR [86], and Venezuela [32]; white plague in Puerto Rico [87]; atramentous necrosis in Australia [29], and white syndrome along the GBR [31]. Of these diseases, three have been identified as caused by a bacterial pathogen resulting in chronic or acute tissue loss: white plague Type II [88], black band disease [89], and white syndrome [90]. The emergent pattern suggests coral diseases that produce progressive tissue loss are responding to seawater temperature whereas those displaying disease signs other than tissue loss are not, or at least not in the same manner. Similarly, we found that the two diseases within CIMR that displayed a positive association with temperature were PorTL and MWS (both tissue loss diseases). Importantly, as only two of our four diseases showed distinct associations with temperature we emphasize that temperature should not be assumed to universally display specific relationships with coral disease prevalence.

A further environmental stressor for coral is reduced water quality, as measured by increased nutrients and chlorophyll-*a* concentration. Reduced water quality has been linked to increased severity and prevalence of aspergillosis in sea fans [33], [35], [37], increased prevalence of yellow band disease [35], and increased black band disease prevalence and progression, respectively [34], [91]. In our study the only diseases to show strong positive associations with increasing chlorophyll-*a* concentration were MWS and, to a lesser degree of predictive importance, PorGA. Consistent with this, MWS prevalence across Kaneohe Bay, an area with historically poor water quality, was found to be four times higher than in other areas of the Main Hawaiian Islands [92].

### Predictor Interactions and Combining Diseases

Researchers often view collinearity between predictor variables as a problem in ecological modeling and remove predictor variables that are highly collineated prior to model formation. However, providing the collinearity between predictors can be identified, quantified and built into the model their synergistic effect may improve model predictive capability. Incorporating interactions between predictor variables increased the predictive power of our models, particularly for *Porites* trematodiasis (PorTrem). When predicting the prevalence of PorTrem, *Porites* cover and overall colony density (the two strongest predictors) were also the two variables showing the highest interaction level (highest degree of departure from a purely additive effect) and together explained the largest amount of variation in the disease occurrence. The number and higher values of the interactions present for PorTrem probably reflects the complex multi-host relations required for this disease to occur. Significant interaction terms between predictors have also been reported for scleractinian coral white syndrome outbreaks in Australia [31] and researchers have started to adopt a more multi-factorial approach to understanding coral disease-environment associations [31], [40]. Our results, in conjunction with these studies, emphasize the need for considering multiple environmental predictors and their respective collinearity for coral disease-environment modeling.

Modeling combined diseases (the prevalence of multiple diseases as a single proportion value as the model response), led to an average six-fold increase in cross-validation deviance (reduction in predictive accuracy) over modeling the diseases individually. This is to be expected. For example, environmental modeling of human cholera (caused by an intestinal bacterium), and measles (a viral disease), even though they affect the same host, would most likely produce confusing results due to their differing etiologies and modes of transmission [13], [71]. However, when disease etiologies are known and their ecological similarities recognized then combined disease modelling may be appropriate. For example, dengue fever and dengue haemorrhagic fever, two diseases both transmitted by mosquitoes within the genus *Aedes*, were modeled together successfully within Thailand [93]. Importantly, the authors were not modeling a combined proportion value of both diseases as the response variable. Modeling overall coral disease prevalence, multiple diseases each with a possibly distinct etiology, seems inappropriate. We recommend coral diseases to be modeled individually, unless they are known to have etiologies that respond in a uniform manner to particular environmental conditions. Predictive statistical modeling forms an important stage in the understanding of coral disease patterns and in conjunction with biomedical techniques, field observations and laboratory manipulations, can increase our understanding of coral disease ecology worldwide.
